# Last resort beta-lactam antibiotics for treatment of New-Delhi Metallo-Beta-Lactamase producing Enterobacterales and other Difficult-to-Treat Resistance in Gram-negative bacteria: A real-life study

**DOI:** 10.3389/fcimb.2022.1048633

**Published:** 2022-12-05

**Authors:** Romaric Larcher, Paul Laffont-Lozes, Claire Roger, Regine Doncesco, Celine Groul-Viaud, Aurelie Martin, Paul Loubet, Jean-Philippe Lavigne, Alix Pantel, Albert Sotto

**Affiliations:** ^1^ Department of Infectious and Tropical Diseases, Nimes University Hospital, Nimes, France; ^2^ PhyMedExp (Physiology and Experimental Medicine), INSERM (French Institute of Health and Medical Research), CNRS (French National Centre for Scientific Research), University of Montpellier, Montpellier, France; ^3^ Department of Pharmacy, Nimes University Hospital, Nimes, France; ^4^ Anesthesiology and Critical Care Medicine, Nimes University Hospital, Nimes, France; ^5^ Department of Microbiology and Hospital Hygiene, Nimes University Hospital, Nimes, France; ^6^ VBIC (Bacterial Virulence and Chronic Infection), INSERM (French Institute of Health and Medical Research), Montpellier University, Nimes, France

**Keywords:** metallo-beta-lactamase, new delhi metallo-beta-lactamase, difficult to treat resistance, pseudomonas aeruginosa, enterobacterales, cefiderocol, ceftazidime-avibactam plus aztreonam, imipenem-relebactam

## Abstract

**Introduction:**

Novel last resort beta-lactam antibiotics are now available for management of infections due to New-Delhi Metallo-Beta-Lactamase (NDM) producing Enterobacterales and non-fermenters with Difficult-to-Treat Resistance. However, data regarding the use of imipenem-cilastatin-relebactam (IMI-REL), cefiderocol (CFD) and ceftazidime-avibactam plus aztreonam (CAZ-AVI-ATM) are scarce in real-life settings. This study aimed to describe the use of last resort beta-lactam antibiotics, the microbiology and the outcome, in patients hospitalized in a tertiary hospital.

**Methods:**

We conducted a monocentric observational cohort study from 2020/01/01, to 2022/08/31. We screened all patients admitted to Nimes University Hospital who have received ≥ 1 dose of last resort beta-lactam antibiotics during the study period, using the Pharmacy database. We included patients treated with IMI-REL, CFD and CAZ-AVI-ATM. The primary endpoint was the infection-free survival rate. We also calculated rates of microbiological and clinical cure, recurrent infection, death and adverse events.

**Results:**

Twenty-seven patients were included in the study and 30 treatment courses were analyzed: CFD (N=24; 80%), CAZ-AVI-ATM (N=3; 10%) and IMI-REL (N=3; 10%). Antibiotics were used in 21 males (70%) and 9 females (30%) with a median age at 65-year-old [50-73.5] and a median Charlson index at 1 [0-2]. Almost all the patients had ≥ 1 risk factor for carbapenem resistant bacteria, a half of them was hospitalized for severe COVID-19, and most of antibiotic courses (N=26; 87%) were associated with ICU admission. In the study population, the probability of infection-free survival at day-90 after last resort beta-lactam therapy initiation was 48.4% CI95% [33.2-70.5]. Clinical failure rate was at 30%, microbiological failure rate at 33% and mortality rate at 23%. Adverse events were documented in 5 antibiotic courses (17%). In details, *P. aeruginosa* were mainly treated with CFD and IMI-REL, *S. maltophilia* with CFD and CAZ-AVI-ATM, *A. baumannii* with CFD, and NDM producing-*K. pneumoniae* with CAZ-AVI-ATM and CFD. After a treatment course with CFD, CAZ-AVI-ATM and IMI-REL, the probability of infection-free survival was 48% CI95% [10.4-73.5], 33.3% CI95% [6.7-100], 66.7% CI95% [30-100], respectively.

**Discussion/conclusion:**

Use of last resort beta-lactam antimicrobials in real-life settings was a safe and efficient therapeutic option for severe infections related to Gram-negative bacteria with Difficult-to-Treat Resistance.

## Introduction

Antimicrobial resistance (AMR), especially in Gram-negative bacteria, is increasing globally and the increasing occurrence of difficult-to-treat infections is resulting in longer hospital stays, higher medical costs, and increased mortality (Antimicrobial Resistance Collaborators, 2022). In this context, the WHO declared AMR is one of the top 10 global health threats, encouraging AMR surveillance, prevention and control efforts and the development of new antimicrobials (World Health Organization, 2016).

Thanks to advance in drugs development, five novel therapeutic options, namely, ceftazidime-avibactam (CAZ-AVI), ceftolozane-tazobactam (TOL-TAZ), meropenem-vaborbactam (MER-VAB), imipenem-cilastatin-relebactam (IMI-REL) and cefiderocol (CFD), have been released during the last decade (Karakonstantis et al., 2020). Moreover, waiting for the release of the novel combination aztreonam-avibactam, the Infectious Diseases Society of America (IDSA) and the European Society of Clinical Microbiology and Infectious Diseases (ESCMID) have recommended the use of ceftazidime-avibactam plus aztreonam (CAZ-AVI-ATM) for infections due to metallo-beta-lactamase type carbapenemase-producing bacteria (Paul et al., 2021; Tamma et al., 2022).

However, data regarding the use of MER-VAB, IMI-REL, CFD and CAZ-AVI-ATM for the treatment of infections due to bacteria with difficult-to-treat resistance (DTR) are scarce in real-life settings (Falcone et al., 2021; Meschiari et al., 2021; Rebold et al., 2021; Falcone et al., 2022).

Thus, the aim of this study was to describe the use of last resort beta-lactam antibiotics, the microbiology and the outcome of patients treated with MER-VAB, IMI-REL, CFD and CAZ-AVI-ATM in a tertiary hospital.

## Materials and methods

### Study design and settings

We carried out a monocentric observational cohort study in the Nimes University Hospital from January 1^st^, 2020, to August 31^st^, 2022. During the study period the intensive care unit (ICU) bed capacity of our 2094-bed teaching hospital increased from 41 to 81 ICU-beds to face the COVID-19 pandemic.

The Institutional Review Board of Nimes University Hospital approved the study (No. 22.07.03) and waived the need for written consent.

### Patients

We screened all consecutive patients hospitalized between January 1^st^, 2020, and May 31^st^, 2022, who have received at least one dose of CFD, IMI-REL, MER-VAB or CAZ-AVI-ATM using the Pharmacy Department database. We reviewed patient medical charts and included adult patients treated at least 72 hours with one of these antibiotics. Patients treated with CAZ-AVI-ATM were included in the study if they were treated according to the international guidelines (Paul et al., 2021; Tamma et al., 2022). When a patient received a last resort beta-lactam antibiotic twice or more, he/she could be included again if the bacteria treated or the antibiotic used was different in subsequent episode. Patients were followed up at least 90 days. Patients aged under 18-year-old and those who did not consent to participate after being informed were excluded.

### Data collection

We collected demographical, clinical and biological data in the digital medical record for each patient. In details, we recorded the date of hospitalization and discharge, the type of antimicrobial therapy received, its dosage regimen and duration, the reason for hospital admission and the type of infection. We also collected the microbiological documentation, the susceptibility of bacteria to antibiotics according to the EUCAST guidelines (The European Committee on Antimicrobial Susceptibility Testing, 2022). Minimal inhibitory concentrations (MICs) of CFD were determined with Sensititre^™^ panel CMP2SHIH or EUSHION8 (ThermoFisher Scientific^™^, Waltham, MA, USA) until January 2022, then with Liofilchem^®^ ComASP^®^ Cefiderocol (Liofilchem^®^, Roseto degli Abruzzi, TE, Italy) broth microdilution panel, MICs of CAZ-AVI-ATM were determined with ETEST^®^ (BioMerieux, Marcy-l’Etoile, France) as previously reported (Emeraud et al., 2019) and MICs of IMI-REL were determined with Liofilchem^®^ MIC Test Strips (MTS). Moreover, the type of carbapenemase was determined as appropriate (GeneXpert Carba-R, Cepheid, USA, CA), and strains of *Pseudomonas aeruginosa* and *Klebsiella pneumoniae* with DTR were sequenced with MiSeq System^®^ using the Nextera^®^ index kit (Illumina^®^, San Diego, CA, USA) then analyzed by whole genome Multilocus Sequencing Typing (wgMLST) with BioMerieux EPISEQ^®^ CS (V1.1). We evaluated comorbid conditions by calculating the Charlson index (Charlson et al., 1994) for each patient, and collected the need for ICU admission and invasive mechanical ventilation. Finally, the vital status at hospital discharge, and 90 days after antimicrobial treatment start was collected.

### Study definitions

We defined clinical failure as the occurrence of death (of any cause), unplanned surgical or percutaneous drainage procedures for complication, or initiation of another antibiotic for worsening symptoms or signs of infection, from start of the initial antibiotic therapy until end of treatment (Musher, 2008).

We defined microbiological failure as growth of the causative pathogen from a blood culture or another sterile site (such as cerebrospinal fluid, empyema, pleural fluid or ascites) at least 5 days from the index culture while the patient was receiving effective antibiotics (Musher, 2008).

Recurrence of infection or recurrent infection refers to a repeat occurrence (second, third or subsequent episode) of infection in a patient, that occurs after the previous/initial episode has been classified as clinically cured (Musher, 2008).

We defined DTR as a bacterial strain intermediate or resistant to all reported agents in carbapenem, β-lactam, and fluoroquinolone categories, including additional agents when results available (Kadri et al., 2018).

The ceftazidime-avibactam standard dosing of 2.5 g (ceftazidime 2g and avibactam 500 mg) plus aztreonam 2g was reported in this study as CAZ-AVI-ATM 2g/0.5g/2g. The imipenem-cilastatin-relebactam standard dosing of 1.25 g (imipenem 500 mg, cilastatin 500 mg, and relebactam 250 mg) was reported in this study as IMI-REL 0.5g/0.25g.

### Statistical analysis

Categorical data were described as numbers and percentages, and continuous data as medians with 25^th^ and 75^th^ percentiles (interquartile range [IQR]). The primary endpoint was the infection-free survival (survival without infection recurrence). Curves of clinical and microbiological success and crude and infection-free survival were obtained by the Kaplan–Meier method. Given the small sample size in this study we did not perform comparative analysis. We performed all statistical analyses with R software, version 4.2.0 (The R Foundation for Statistical Computing, Vienna, Austria).

## Results

### Population

**Figure 1:**
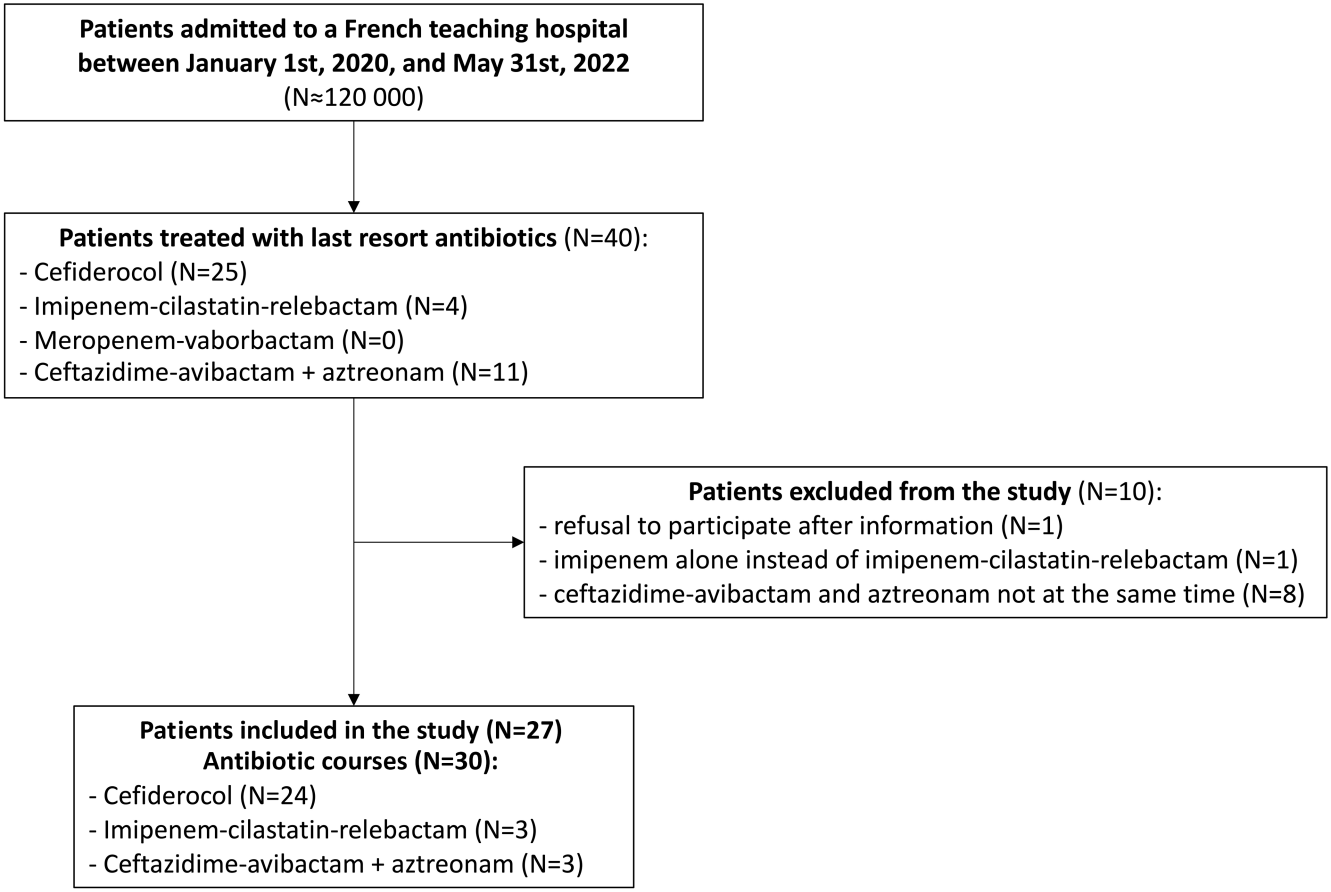
Flowchart.

About 120 000 patients were admitted to our hospital during the study period. Of them, only 27 received last resort beta-lactam antibiotics, accounting for 30 treatment courses (**Figure 1**). Cefiderocol accounted for 24 antibiotic courses (80%), CAZ-AVI-ATM and IMI-REL were used in only 3 patients (10%), respectively, while no patient had MER-VAB. One patient received CFD then CAZ-AVI-ATM, one received CFD then IMI-REL and one received CFD two times at a different time during hospital stay.

The characteristics of the study population are summarized in **Table 1**. Antibiotics were used in 21 males (70%) and 9 females (30%) with a median age at 65-year-old [50-73.5] and a median Charlson index at 1 [0-2]. More than three quarter of patients were treated with carbapenem within a month before the onset of infection requiring last resort beta-lactam antibiotics. Patients were mainly admitted for low respiratory tract infection, especially, a half of the patient was hospitalized for severe COVID-19. During their hospital stay, most of the patients (N=26; 87%) were admitted to the ICU and 21 (70%) required invasive mechanical ventilation. All but one of the patients were treated with antibiotics for a median duration of 22.5 days [11-35] between admission and infection onset requiring last resort beta-lactam antibiotic.

**Table 1:** Characteristics of the study population.

	Last resort beta-lactams (N=30) N (%) or median [IQR]	CFD (N=24) N (%) or median [IQR]	CAZ-AVI-ATM (N=3) N (%) or median [IQR]	IMI-REL (N=3) N (%) or median [IQR]
Male	22 (73%)	18 (75%)	2 (67%)	2 (67%)
Age (year-old)	65 [50-73.5]	65 [50-71.5]	74 [74-76.5]	50 [50-61]
BMI (kg/m^2^)	30 [26.5-31]	28.8 [26.5-31]	32 [31.8-41]	30 [30-30]
Creatinine (µmol/L)	51 [33-114]	47 [31-102]	114 [87-154]	39 [39-150]
Charlson index	1 [0-2]	2 [0-2]	1 [1-2]	0 [0-3]
**Risk factors for carbapenem resistance**				
Hospitalization in the last 6 months	13 (43%)	9 (38%)	1 (33%)	3 (100%)
in another hospital	7 (23%)	6 (25%)	1 (33%)	0 (0%)
Recent trip in a foreign country	1 (3%)	1 (4%)	0 (0%)	0 (0%)
SOT in the last 3 months	3 (10%)	2 (8%)	0 (0%)	1 (33%)
Chemotherapy in the last 3 months	1 (3%)	1 (4%)	0 (0%)	0 (0%)
Corticosteroids in the last 3 months	1 (3%)	1 (4%)	0 (0%)	0 (0%)
Surgery in the last month	9 (30%)	7 (29%)	1 (33%)	1 (33%)
Carbapenem in the last month	23 (77%)	18 (75%)	1 (33%)	2 (67%)
**Reason for admission**				
Surgery	7 (23%)	6 (25%)	0 (0%)	1 (33%)
Unscheduled	3 (10%)	3 (13%)	0 (0%)	0 (0%)
Scheduled	4 (13%)	3 (13%)	0 (0%)	1 (33%)
Medical	23 (77%)	18 (75%)	3 (100%)	2 (67%)
Infection	27 (90%)	21 (87%)	3 (100%)	3 (100%)
LRTI	16 (53%)	12 (50%)	2 (67%)	2 (67%)
COVID-19	15 (50%)	12 (50%)	2 (67%)	1 (33%)
IAI	5 (17%)	5 (21%)	0 (0%)	0 (0%)
BJI	3 (10%)	1 (4%)	1 (33%)	1 (33%)
SSTI	2 (7%)	2 (8%)	0 (0%)	0 (0%)
Meningitis	1 (3%)	1 (4%)	0 (0%)	0 (0%)
Trauma	3 (10%)	3 (13%)	0 (0%)	0 (0%)
**Management**				
HLOS (days)	79 [50-117]	84 [55-123]	48 [37-65]	117 [97-117]
ICU admission	26 (87%)	22 (92%)	3 (100%)	1 (33%)
Mechanical ventilation	21 (70%)	17 (71%)	2 (67%)	2 (67%)
Adequate source control	29 (97%)	23 (96%)	3 (100%)	3 (100%)
**Antibiotics received during hospital stay**				
≥ 1 antibiotic	29 (97%)	23 (96%)	3 (100%)	3 (100%)
Duration (days)	22.5 [11-35]	29 [11.5-37]	11 [10.5-20.5]	17 [14.5-26]
Piperacillin-tazobactam	15 (50%)	12 (50%)	1 (33%)	2 (67%)
Cefepime	17 (57%)	15 (63%)	2 (67%)	0 (0%)
Carbapenem	18 (60%)	16 (60%)	1 (33%)	1 (33%)
Ceftazidime-avibactam	4 (13%)	4 (17%)	0 (0%)	0 (0%)
Cefiderocol	3 (10%)	1 (4%)	1 (33%)	1 (33%)
Linezolid	13 (43%)	10 (42%)	2 (67%)	1 (33%)
Vancomycin	6 (20%)	4 (17%)	1 (33%)	1 (33%)
Daptomycin	1 (3%)	0 (0%)	1 (33%)	0 (0%)
Metronidazole	3 (10%)	3 (13%)	0 (0%)	0 (0%)
Fluoroquinolone	7 (23%)	6 (25%)	0 (0%)	1 (33%)
Cotrimoxazole	4 (13%)	3 (13%)	1 (33%)	0 (0%)
Macrolide	2 (7%)	2 (8%)	0 (0%)	0 (0%)
Tigecycline	1 (3%)	1 (4%)	0 (0%)	0 (0%)
Colistin	2 (7%)	2 (8%)	0 (0%)	0 (0%)
Fosfomycin	1 (3%)	1 (4%)	0 (0%)	0 (0%)
Aminoglycoside	2 (7%)	2 (8%)	0 (0%)	0 (0%)

BJI, bone and joint infection; BMI, body mass index; CAZ-AVI-ATM, ceftazidime-avibactam-aztreonam; CFD, cefiderocol; HLOS, hospital length of stay; IAI, intra-abdominal infection; ICU, intensive care unit; IMI-REL, imipenem-relebactam; IQR, interquartile range; LRTI, low respiratory tract infection; SSTI, skin and soft tissue infection; SOT, solid organ transplant.

### Antimicrobial therapies and outcomes

The characteristics of last resort beta-lactam antimicrobial therapies are shown in **Table 2**.

**Table 2:** Characteristics of last resort beta-lactam antimicrobial therapies.

	Last resort antibiotics (N=30) N (%) or median [IQR]	CFD (N=24) N (%) or median [IQR]	CAZ-AVI-ATM (N=3) N (%) or median [IQR]	IMI-REL (N=3) N (%) or median [IQR]
**Infection site**				
Pneumonia	21 (70%)	17 (71%)	2 (67%)	2 (67%)
VAP	19 (63%)	17 (71%)	1 (33%)	1 (33%)
Bloodstream infection	6 (20%)	5 (21%)	1 (33%)	0 (0%)
CR-BSI	4 (13%)	3 (13%)	1 (33%)	0 (0%)
BJI	3 (10%)	2 (8%)	0 (0%)	1 (33%)
IAI	2 (7%)	2 (8%)	0 (0%)	0 (0%)
UTI	1 (3%)	1 (4%)	0 (0%)	0 (0%)
Meningitis	1 (3%)	1 (4%)	0 (0%)	0 (0%)
**Reason for antibiotic initiation**				
DTR *P. aeruginosa*	21 (70%)	19 (79%)	0 (0%)	3 (100%)
DTR *S. maltophilia*	2 (7%)	2 (8%)	0 (0%)	0 (0%)
DTR *A. xylosoxidans*	1 (3%)	1 (4%)	0 (0%)	0 (0%)
CRAB	1 (3%)	1 (4%)	0 (0%)	0 (0%)
NDM *K. pneumoniae*	4 (13%)	1 (4%)	3 (100%)	0 (0%)
ESAC *K. aerogenes*	1 (3%)	1 (4%)	0 (0%)	0 (0%)
ESAC *E. cloacae*	2 (7%)	1 (4%)	1 (33%)	0 (0%)
**Antimicrobial therapy management**				
Duration (days)	14,5 [12-37]	14 [12-31]	14 [12-18]	42 [35-42]
Daily dose (g)	–	6	6/1.5/6	2/1
Daily regimen	–	TID	TID	QID
Infusion time (h)	–	3 [3-4]	2 [2-7]	0,5 [0,5-0,5]
Combination therapy	18 (60%)	13 (54%)	3 (100%)	2 (67%)
fluoroquinolone	5 (17%)	5 (21%)	0 (0%)	0 (0%)
cotrimoxazole	2 (7%)	0 (0%)	2 (66%)	0 (0%)
fosfomycin	2 (7%)	1 (4%)	0 (0%)	1 (33%)
colistin	1 (3%)	0 (0%)	0 (0%)	1 (33%)
tigecycline	1 (3%)	0 (0%)	1 (33%)	0 (0%)
linezolid	4 (13%)	2 (8%)	1 (33%)	1 (33%)
vancomycin	2 (7%)	2 (8%)	0 (0%)	0 (0%)
metronidazole	1 (3%)	1 (4%)	0 (0%)	0 (0%)
**Reason for antibiotic cessation**				
End of cure	18 (60%)	13 (54%)	1 (33%)	3 (100%)
De-escalade	2 (7%)	2 (8%)	0 (0%)	0 (0%)
Treatment failure	10 (33%)	8 (33%)	2 (67%)	0 (0%)
**Global outcomes**				
Death (all cause)	7 (23%)	5 (21%)	2 (67%)	0 (0%)
Sepsis-related	4 (13%)	3 (13%)	1 (33%)	0 (0%)
WLS	3 (10%)	2 (8%)	1 (33%)	0 (0%)
Microbiological failure	10 (33%)	10 (42%)	0 (0%)	0 (0%)
Clinical failure	9 (30%)	7 (29%)	2 (66%)	0 (0%)
Recurrent infection	6 (20%)	5 (21%)	0 (0%)	1 (33%)
Adverse event*	5 (17%)	3 (13%)	1 (33%)	1 (33%)

BJI, bone and joint infection; CAZ-AVI-ATM, ceftazidime-avibactam-aztreonam; CFD, cefiderocol; CR-BSI, catheter-related bloodstream infection; CRAB, carbapenem resistant Acinetobacter baumannii; DTR, difficult to treat resistance; ESAC, extended-spectrum AmpC beta-lactamase; IAI, intra-abdominal infection; IMI-REL, imipenem-relebactam; LRTI, low respiratory tract infection; NDM, New-Delhi Metallo-beta-lactamase; SSTI, skin and soft tissue infection; UTI, urinary tract infection; VAP, ventilator associated pneumonia; WLS, withdrawal of life support. *Details of adverse event, eosinophilia (cefiderocol N=1, imipenem-relebactam N=1), diarrhea (cefiderocol N=1), hepatitis (cefiderocol N=1).

Last resort beta-lactam antibiotics were mainly initiated for treatment of pneumonia (N=21; 70%), followed by bloodstream infections (N=6; 20%), bone and joint infections (N=2), intra-abdominal infections (N=2), urinary tract infection (N=1) and meningitis (N=1). The use of CFD and IMI-REL was mainly driven by positive culture results for DTR-*P. aeruginosa*, whereas the use of CAZ-AVI-ATM was related to positive bacteriological samples for New-Delhi Metallo-Beta-Lactamase (NDM)-1 producing *K. pneumoniae* (N=4; 13%). More than a half of patient had a combination therapy (N=18; 60%). Of note, CFD and CAZ-AVI-ATM were always administered by extended or continuous infusion whereas IMI-REL was administered by intermittent infusion.

Last resort beta-lactam antibiotics were stopped at the end of cure in 18 patients (60%) and none of the 5 adverse events (17%) were responsible for treatment cessation. A de-escalade was done in two patients after 72 hours (CFD for piperacillin-tazobactam) and 96 hours (CFD for cefepime plus ciprofloxacin), respectively. Less than a third of antibiotic treatment results in clinical failure, including seven deaths (23%) related to sepsis (N=4), and withdrawal of life support (N=3). The crude mortality rate among patients included in the analysis was 26% (7/27). Microbiological failure was reported in 10 patients (33%) and 5 patients (17%) had infection recurrence.

Importantly, among microbiological failures, two were related to an acquisition of CFD resistance in *P. aeruginosa* isolates during treatment with this antibiotic (MIC increased from 1 to 8 mg/L and 1 to 4 mg/L, respectively), and led to clinical failure in both patients (21 and 25), but none died. In one of these patients, wgMLST has highlighted that first strains susceptible to CFD had numerous *Pseudomonas* derived cephalosporinase (PDC) alleles (namely, *bla*_PDC-3_, *bla*_PDC-176_, *bla*_PDC-191_, *bla*_PDC-192_ and *bla*_PDC-272_). On the contrary, in strains resistant to CFD, only one PDC allele was identified (*bla*_PDC-392_ or *bla*_PDC-394_).

**Figure 2:**
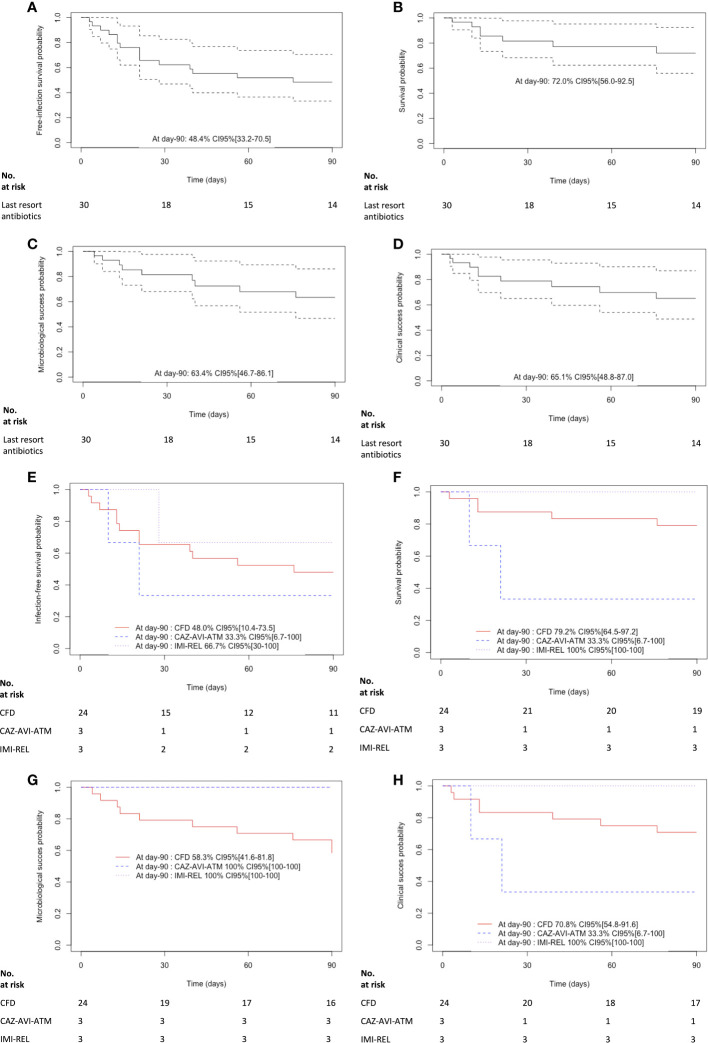
Kaplan-Meier curves of **(A)** the infection free survival in patients treated with last resort beta-lactam antibiotics. The dashed lines represent the 95% confidence interval, **(B)** the survival in patients treated with last resort beta-lactam antibiotics. The dashed lines represent the 95% confidence interval, **(C)** the microbiological cure in patients treated with last resort beta-lactam antibiotics. The dashed lines represent the 95% confidence interval, **(D)** the clinical cure in patients treated with last resort beta-lactam antibiotics. The dashed lines represent the 95% confidence interval, **(E)** the infection free survival in patients treated with cefiderocol (CFD), imipenem-cilastatin-relebactam (IMI-REL) and ceftazidime-avibactam plus aztreonam (CAZ-AVI-ATM), **(F)** the survival in patients treated with CFD, IMI-REL and CAZ-AVI-ATM, **(G)** the microbiological cure in patients treated with CFD, IMI-REL and CAZ-AVI-ATM, and **(H)** the clinical cure in CFD, IMI-REL and CAZ-AVI-ATM.

Globally, in the study population, the probability of infection-free survival was 48.4% CI95% [33.2-70.5] at day-90 after last resort beta-lactam antimicrobial therapy initiation (**Figure 2**). The probability of infection-free survival was 48% CI95% [10.4-73.5], 33.3% CI95% [6.7-100], 66.7% CI95% [30-100], after CFD, CAZ-AVI-ATM and IMI-REL, respectively (**Figure 2**).

Details on antimicrobial therapy, microbiology and outcomes are presented in **Tables 3**, **4**.

**Table 3:** Rates of bacterial strain susceptible to carbapenem and second- and third-line beta-lactam antibiotics and free-infection survival rates after antimicrobial therapy for difficult-to-treat bacteria.

Microorganism	Antimicrobial susceptibility testing (susceptible/tested)								Free-infection survival rates		
	IMI	MER	CAZ-AVI	TOL-TAZ	MER-VAB	IMI-REL	CAZ-AVI-ATM	CFD	IMI-REL	CAZ-AVI-ATM	CFD
*P. aeruginosa*	25% (6/24)	17% (4/24)	61% (14/23)	73% (16/22)	47% (8/17)	59% (10/17)	100% (2/2)	95% (21/22)			
*DTR*	*5% (1/21)*	*0% (0/21)*	*57% (12/21)*	*67% (14/21)*	*36% (5/14)*	*50% (7/14)*	–	*95% (18/19)*	67% (2/3)	–	58% (11/19)
*S. maltophilia*	0% (0/5)	0% (0/5)	0% (0/3)	–	–	–	50% (1/2)	100% (4/4)		50% (1/2)	25% (1/4)
*A. baumannii*	0% (0/1)	0% (0/1)	0% (0/1)	0% (0/1)	0% (0/1)	0% (0/1)	–	100% (1/1)	–	–	0% (0/1)
*A. xylosoxidans*	100% (1/1)	0% (0/1)	0% (0/1)	0% (0/1)	0% (0/1)	–	–	0% (0/1)	–	–	100% (1/1)
*Enterobacterales*	67% (8/12)	67% (8/12)	73% (8/11)	40% (4/10)	75% (9/12)	73% (8/11)	100% (5/5)	73% (8/11)			
*ESAC*	*66% (2/3)*	*66% (2/3)*	*100% (0/3)*	*0% (0/1)*	*100% (3/3)*	*100% (2/2)*	*100% (2/2)*	*33% (1/3)*	–	0% (1/1)	0% (0/2)
*NDM*	*0% (0/3)*	*0% (0/3)*	*0% (0/3)*	*0% (0/3)*	*0% (0/3)*	*0% (0/3)*	*100% (3/3)*	*33% (1/3)*	–	33% (1/3)	0% (0/1)
Total	33% (14/42)	26% (11/42)	56% (22/39)	59% (20/34)	55% (17/31)	62% (18/29)	100% (8/8)	97% (34/39)			

ATM-AVI, aztreonam-avibactam; BJI, bone and joint infection; BSI, bloodstream infection; CAZ-AVI, ceftazidime-avibactam; CR-BSI, catheter-related BSI; DTR, difficult to treat; IAI, intraabdominal infection; IMI, imipenem; IMI-REL, imipenem-relebactam; MER, meropenem; MER-VAB, meropenem-vaborbactam; MIC, minimum inhibitory concentration, NDM, New-Delhi Metallo-beta-lactamase; TOL-TAZ, ceftolozane-tazobactam; VAP, ventilator associated pneumonia.

**Table 4:** Details of antimicrobial therapies, microbiology and outcomes.

#	Infection sites	Microorganisms	Antimicrobial susceptibility testing (MIC)								Antibiotics regimen	Duration (days)	Outcomes
		(Sequencing Type)	IMI	MER	CAZ-AVI	TOL-TAZ	MER-VAB	IMI-REL	CAZ-AVI-ATM	CFD			
1	LRTI	*S. maltophilia* (cotrimoxazole-R, levofloxacin-R)	R	R	R	–	–	–	–	S (0.5)	CFD	76	Microb. failure
	(VAP)										2g over 4h		Clinical failure
											BID		Dead
											(CVVHD)		
		
2	UTI	*P. aeruginosa* (ST175)	R	R	R	S	–	R	–	S (2.0)	CFD	4	Microb. cure
	IAI										2g over 3h		Clinical cure
		*P. aeruginosa*	S	S	–	–	–	–	–	–	TID		Alive
													De-escalade
											
3	LRTI	*P. aeruginosa* (ST1613)	R	R	R	R	R	R	–	S (0.5)	CFD	21	Microb. cure
	(VAP)										2g over 4h		Clinical cure
											TID		Alive
													Recurrence
		
4	BJI	*P. aeruginosa*	S	R	R	R	–	S	–	S (1.0)	CFD	28	Microb. cure
											0.5g over 3h		Clinical cure
											TID		Alive
	
5	LRTI	*P. aeruginosa*	R	R	S	S	R	R	–	S (1.0)	CFD	12	Microb. cure
	(VAP)										0.75g over 3h		Clinical cure
		*K. pneumoniae*	S	S	S	S	S	S	–	S	BID		Alive
											(+ciprofloxacin)		
		*C. koseri*	S	S	S	S	S	S	–	S			
											
6	LRTI	*P. aeruginosa* (ST2128)	R	R	R	R	R	R	–	S (2.0)	CFD	90	Microb. failure
	(VAP)										2g over 4h		Clinical cure
	BSI	*K. pneumoniae*	S	S	S	S	S	S	–	S	QID		Alive
	(CR-BSI)												
											
7	IAI	*P. aeruginosa* (ST309)	R	R	S	S	S	S	–	S (1.0)	CFD	7	Microb. failure
											2g over 3h		Clinical cure
											TID		Alive Recurrence
	
8	Meningitis	*P. aeruginosa* (ST309)	R	R	R	R	S	R	–	S (1.0)	CFD	21	Microb. cure
											2g over 4h		Clinical cure
											6 times a day		Alive
	
9	BSI	*P. aeruginosa* (ST313)	R	R	S	S	R	R	–	S (0.5)	CFD	12	Microb. cure
	(CR-BSI)										2g over 4h		Clinical cure
		*K. pneumoniae*	S	S	S	S	S	S	–	S	TID		Alive
											(+ciprofloxacin)		
		*S. maltophilia*	R	R	R	–	–	–	–	S			
											
10	BJI	*P. aeruginosa* (ST244)	S	S	S	S	S	S	–	S	CFD	3	Microb. cure
	BSI										2g over 3h		Clinical cure
											TID		Alive
													De-escalade
		
11	LRTI	*A. baumannii*	R	R	R	R	R	R	–	S (0.5)	CFD	3	Microb. cure
	(VAP)										2g over 4h		Clinical failure
	BSI										TID		Dead
		
12	LRTI	*S. maltophilia* (cotrimoxazole-R)	R	R	R	–	–	–	R (24.0)	S (1.0)	CFD	13	Microb. failure
	(VAP)	*E. cloacae*									1.5g over 3h		Clinical failure
	BSI		S	S	S	R	S	S	–	S	TID		Dead
											(+levofloxacin)		
											
13	LRTI	*P. aeruginosa* (ST2996)	R	R	R	S	–	–	–	S	CFD	14	Microb. cure
	(VAP)										2g over 3h		Clinical cure
		*K. pneumoniae*	S	S	–	–	S	S		S	TID		Alive
										
14	LRTI	*P. aeruginosa* (ST313)	R	R	S	S	S	–	–	S (0.5)	CFD	15	Microb. cure
	(VAP)										2g over 4h		Clinical cure
		*A. xylosoxidans*	S	R	R	R	R	–	–	R	QID		Alive
											
15	LRTI	*P. aeruginosa*	R	R	S	S	–	–	–	S	CFD	15	Microb. cure
	(VAP)										2g over 3h		Clinical cure
	BSI										TID		Alive
											(+ciprofloxacin)		
#	Infection sites	Microorganisms	Antimicrobial susceptibility testing (MIC)								Antibiotics	Duration (days)	Outcomes
			IMI	MER	CAZ-AVI	TOL-TAZ	MER-VAB	IMI-	CAZ-AVI-ATM	CFD			
								REL					
16	LRTI	*P. aeruginosa* (ST308)	R	R	S	S	–	S (1.0)	–	S (0.5)	CFD	21	Microb. failure
	(VAP)										2g over 3h		Clinical cure
											TID		Alive
													Recurrence
		
											CFD	39	Microb. failure
											2g over 3h		Clinical failure
											TID		Dead
											(+fosfomycin)		
		
17	LRTI	*K. aerogenes*	R	R	S	R	S	–	–	S (0.5)	CFD	14	Microb. failure
	(VAP)										2g over 3h		Clinical cure
											TID		Alive
											(+cotrimoxazole)		Recurrence
		
18		*P. aeruginosa* (ST2128)	R	R	R	S	R	R	–	S (1.0)	CFD	14	Microb. cure
	LRTI										2g over 3h		Clinical cure
	(VAP)										TID		Alive
		
19		*P. aeruginosa* (ST654)	R	R	S	S	R	–	–	S (0.5)	CFD	13	Microb. cure
	LRTI										2g over 3h		Clinical failure
	(VAP)										TID		Dead
		
20	LRTI	*P. aeruginosa*	R	R	R	R	–	–	–	S	CFD	14	Microb. cure
	(VAP)										2g over 3h		Clinical cure
		*P. aeruginosa*	R	R	S	S	–	–	–	S	TID		Alive
											
21	LRTI	*P. aeruginosa* (ST1613)	R	R	S	S	S	S	–	S (1.0)	CFD	40	Microb. failure
	(empyema)										2g over 3h		Clinical cure
											TID		Alive
													Recurrence
		
22	LRTI	*K. pneumoniae* (ST147)	R	R	R	R	R	R	S (0.38)	S (2.0)	CFD	4	Microb. failure?
	(VAP)	*E. cloacae*									0.75g over 3h		Clinical failure
	BSI		S	S	S	–	S	S	S (1.0)	R (4.0)	BID		Alive
		*P. aeruginosa*											
			S	S	S	–	S	S	S	S			
		*S. maltophilia*											
			R	R	–	–	–	–	–	S	CAZ-AVI-ATM	21	Microb. cure
											2g/0.5g/2g		Clinical failure
											over 2h TID		Dead
											(+cotrimoxazole)		
23	BSI	*K. pneumoniae* (ST247)	R	R	R	R	R	R	S (1.5)	R		10	Microb. cure
	(CR-BSI)										CAZ-AVI-ATM		Clinical failure
											2g/0.5g/2g		Dead
											over 2h TID		
											(+tigecycline)		
		
24	LRTI	*K. pneumoniae* (ST147)	R	R	R	R	R	R	S (0.12)	R	CAZ-AVI-ATM	14	Microb. cure
									S		1g/0.25g/1g		Clinical cure
		*K. pneumoniae*	S	S	S	R	S	S		–	over 12h BID		Alive
									S				
		*P. aeruginosa*	S	S	S	S	S	S		–			
									S				
		*S. maltophilia*	R	R	–	–	–	–		–			
										
25	LRTI	*P. aeruginosa* (ST679)	S	R	R	R	R	S (0.75)	–	S (1.0)	CFD	56	Microb. failure
	(VAP)										2g over 6h		Clinical failure
											QID		Alive
											(+ciprofloxacin)		
		
											IMI-REL	35	Microb. cure
											1g/0.25g		Clinical cure
											over 0.5h TID		Alive
											(+colistin		
											+fosfomycin)		
		
26	BJI	*P. aeruginosa*	R	R	S	S	R	S (2.0)	–	S (1.0)	IMI-REL	28	Microb. cure
											0.5g/0.25g		Clinical cure
											over 0.5h BID		Alive
													Recurrence
27	LRTI	*P. aeruginosa* (ST1613)	R	R	S	S	S	S (0.5)	–	R (4.0)	IMI-REL	42	Microb. cure
	(empyema)										0.5g/0.25g		Clinical cure
											over 0.5h QID		Alive

BID: two times a day; BJI: bone and joint infection; BSI: bloodstream infection; CAZ-AVI: ceftazidime-avibactam; CAZ-AVI-ATM: ceftazidime-avibactam-aztreonam; CFD: cefiderocol; CR-BSI: catheter-related bloodstream infection; IAI: intraabdominal infection; IMI: imipenem; IMI-REL: imipenem-relebactam; LRTI: low respiratory tract infection; MER: meropenem; MER-VAB: meropenem-vaborbactam; MIC: minimum inhibitory concentration, Microb.: microbiological; QID: four times a day; TID: three times a day; TOL-TAZ: ceftolozane-tazobactam; VAP: ventilator associated pneumonia, #: patient number

More than one quarter of the patients had polymicrobial infection (N=8; 27%). Most of the patients were treated with CFD for infections related to DTR-*P. aeruginosa* highlighting a free-infection survival rate at 58%. Of them, two patients were successfully treated for a BJI and one for a nosocomial meningitis. Moreover, a patient with pneumonia related to DTR-*P. aeruginosa* and *Achromobacter xylosoxidans* resistant to CFD had a favorable outcome after two-week of CFD. On the contrary, infections treated with CFD and related to Enterobacterales and/or other non-fermenters such as *Stenotrophomonas maltophilia* or carbapenem-resistant-*Acinetobacter baumannii* (CRAB) showed lower free-infection survival rates (0-25%). It is noteworthy that one of three NDM-1-producing *K. pneumoniae* was susceptible to CFD whereas an ESAC-producing *E. cloacae* was resistant (MIC 4 mg/L). Interestingly, IMI-REL was not used for the treatment of carbapenem-resistant-Enterobacterales (CRE) but exclusively for infections due to DTR-*P. aeruginosa* (including a BJI), and showed a free-infection survival rate at 66%. Finally, CAZ-AVI-ATM was used in 3 patients infected with NDM-1-producing *K. pneumoniae*. Two of them had polymicrobial infections, with an extended-spectrum AmpC β-lactamase (ESAC)-producing *Enterobacter cloacae* and an extended-spectrum β-lactamase (ESBL)-producing *K. pneumoniae*. The free-infection survival rate was at 33% in patients who received CAZ-AVI-ATM.

## Discussion

We reported herein the results of a cohort study that included 27 patients hospitalized in a teaching hospital in France, treated with last resort beta-lactam antimicrobial therapies for severe infections related to Gram-negative bacteria with DTR. Cefiderocol and IMI-REL were mainly used for treatment of DTR-non fermenters, whereas NDM-producing Enterobacterales were treated with CAZ-AVI-ATM. Although we reported clinical and microbiological success in around two thirds of 30 antibiotic courses, less than a half resulted in infection-free survival at day-90.

In accordance with our results, during the COVID-19 pandemic, most of the resistance was carried in Gram-negative bacteria such as *P. aeruginosa* and *K. pneumoniae* and in patients requiring invasive ventilation (The coVAPid study Group et al., 2021; Kariyawasam et al., 2022). Moreover the prognosis in our cohort was also in line, with that reported in infections due to bacteria with DTR in which around a half of the patients had unfavorable outcomes (Kadri et al., 2018; Giannella et al., 2019; Strich and Kadri, 2019; Bassetti et al., 2020).

Ceftazidime-avibactam and TOL-TAZ are first line agent for management of DTR-*P. aeruginosa* infections (Balandin et al., 2021; Gill et al., 2021; Sader et al., 2021). Unfortunately, from December 2020 to February 2022, TOL-TAZ was recalled from all markets worldwide. Consequently, alternative such as CFD have been proposed (Meschiari et al., 2021). In patient infected with DTR- *P. aeruginosa* treated with CFD, we found a 63% rate of microbiological cure and a 79% rate of clinical cure, in accordance with rates (70.6% and 76.5%, respectively) previously reported (Meschiari et al., 2021). Moreover, we used CFD with success in CAZ-AVI- and/or TOL-TAZ-resistant-*P. aeruginosa* infections, which confirmed its role for the treatment of the more resistant species of *P. aeruginosa* (Bassetti et al., 2020; Syed, 2021).

One interesting finding in our study was the acquisition of CFD resistance in *P. aeruginosa* isolates which has led to clinical failure in two patients during treatment with this antibiotic. A high prevalence of heteroresistance to CFD has been proposed as an explanation for multifold increases in CFD MICs and CFD treatment failure against carbapenem-resistant bacteria, but reliable clinical data are still lacking (Choby et al., 2021a; Choby et al., 2021b). Cefiderocol resistant *P. aeruginosa* strains are usually attributed to metallo-beta-lactamase (MBL) or *Pseudomonas* extended resistant β-lactamase (PER) production which was not found in our cases (Yamano, 2019). As suggested by wgMLST in the present study, some authors have also proposed CFD resistance in *P. aeruginosa* could be related to substitutions in the region of the AmpC omega loop (Simner et al., 2021). Thus, a single amino acid substitution has the potential to inactive TOL-TAZ, CAZ-AVI, and CFD) while potentially increasing activity of IMI-REL (Simner et al., 2021). In our cases, one of the two stains of *P. aeruginosa* resistant to CFD remained susceptible to TOL-TAZ and CAZ-AVI (and IMI-REL), underlining CFD resistance is a complex phenomenon not well characterized and in need of continued exploration (McCreary et al., 2021).

Originally developed for the treatment of class A beta-lactamases *Klebsiella pneumoniae* carbapenemase (KPC) producing Enterobacterales, IMI-REL has rapidly shown its potential role in the treatment of DTR-*P. aeruginosa* infections (Lob et al., 2019; Motsch et al., 2020; Mushtaq et al., 2021a). As a matter of fact, loss of OprD only confers resistance to imipenem if PDC is expressed, thus, the potentiation of imipenem by relebactam is mainly related to its ability to protect imipenem from derepression of AmpC, but also from up-regulation of efflux (Horner et al., 2019). Accordingly, a large number of DTR-*P. aeruginosa* isolates (67%) remained susceptible to IMI-REL in our cohort. Some authors (Boulant et al., 2019) have suggested that the occurrence of resistance to TOL-TAZ could restore the susceptibility to IMI-REL while only 2 of 5 (40%) *P. aeruginosa* isolates resistant to TOL-TAZ were susceptible to IMI-REL in our study. Once again, this result emphasized the extraordinary capacity of *P. aeruginosa* to confer resistance *via* multiple mechanisms, involving AmpC derepression, loss of OprD, up-regulation of efflux, and sometimes MBL production (Lob et al., 2021). Importantly, of the 3 patients treated with IMI-REL for DTR-*P. aeruginosa* infections in our case series, all had microbiological and clinical cures, including one patient with bone infection (which had a recurrent bone infection with a different strain of *P. aeruginosa*).

Among due to antimicrobial-resistant bacteria infections, those related to carbapenem-resistant Enterobacterales remains the most challenging to manage (Mojica et al., 2022). Since the release of CAZ-AVI, and later IMI-REL and MER-VAB, treatment options are available for non-carbapenemase-producing-carbapenem-resistant-Enterobacterales (non-CPE-CRE), and class A beta-lactamases KPC producing Enterobacterales infections (Senchyna et al., 2019). In addition, CAZ-AVI is also effective against isolates harboring class D beta-lactamases such as Oxacillinase-48 (OXA-48-like) carbapenemases producing Enterobacterales. Recently, the release of CFD offered a novel therapeutic option for infections related to MBL producers (Timsit et al., 2022a). Cefiderocol is relatively stable to MBL such as imipenemase (IMP) or Verona imipenemase (VIM) enzymes, however, the MICs for Enterobacterales (and non-fermenters) with NDM carbapenemases tend to be higher (Boyd et al., 2020). In accordance, clinical cure reported in the literature was lower for NDM-producing Enterobacterales (56.2%) than for other CRE (100%) infections (Timsit et al., 2022a).

In our study, two NDM producing-*K. pneumoniae* strains were resistant to CFD and one strain was susceptible to this antibiotic. The patient with the strain susceptible was treated with CFD, however, the antibiotic was stopped after 4 days because of microbiological and clinical failure related to bloodstream infection documented with ESAC-producing *E. cloacae* resistant to CFD. It is noteworthy, that rapid acquisition of CFD resistance in *E. cloacae* through mutations of the CirA siderophore receptor during CFD therapy have been recently reported in the literature (Klein et al., 2022; Nurjadi et al., 2022). It has also been suggested that acquisition of CFD resistance during treatment could be related to overproduction of NDM enzymes (Johnston et al., 2020), mutations affecting porins and efflux pumps, mutations in penicillin-binding-protein 3, and heteroresistance mechanisms (Karakonstantis et al., 2022; Witt et al., 2022).

For the treatment of bacteremia and pneumonia due to MBL producing-Enterobacterales, CAZ-AVI-ATM combination has shown promising result (Falcone et al., 2021; Timsit et al., 2022b). Three patients received CAZ-AVI-ATM in our cohort for NDM-producing *K. pneumoniae* infections. Two of them died of bloodstream infection (pneumonia- and catheter-related, respectively) and the last recovered from a pneumonia. Since the use of CFD remains debated in NDM producing-Enterobacterales infections, CAZ-AVI-ATM is now considered as the first line therapeutic option in this indication (Klein et al., 2022; Nurjadi et al., 2022; Timsit et al., 2022a). Indeed, the risk of resistance acquisition to aztreonam-avibactam appears to be relatively small in NDM producing-Enterobacterales infections (Mushtaq et al., 2021b). On the contrary, CFD could be more suitable than CAZ-AVI-ATM for the treatment of MBL- producing non-fermenters such as *P. aeruginosa* (Delgado-Valverde et al., 2020; Lee et al., 2021; Mauri et al., 2021).

In the same way, *S. maltophilia* that has two intrinsic chromosomal inducible beta-lactamases (L1, a metallo-beta-lactamase and L2, a serine-cephalosporinase) is resistant to carbapenem and most of time to all β-lactam therapies (Gibb and Wong, 2021). Avibactam has been reported to be able to inhibit *S. maltophilia* beta-lactamases activity in order to restore the activity of aztreonam, and in a lesser extend of ceftazidime (Mojica et al., 2017). Both antimicrobial therapies, namely CAZ-AVI-ATM and especially CFD, are promising option, however clinical data are limited (Bassetti et al., 2020; Gibb and Wong, 2021). Our results showed that 50% (1/2) of *S. maltophilia* strains tested were susceptible to CAZ-AVI-ATM and 100% (4/4) were susceptible to CFD. In addition, 50% (1/2) of patients treated with CAZ-AVI-ATM had favorable outcomes whereas those treated with CFD had 25% (1/4) of favorable outcomes. Clinical evidence is awaited to determine which is the best option for the treatment of *S. maltophilia*, especially for extensively drug resistant strains (Gibb and Wong, 2021).

Only one patient received CFD for a CRAB related infection and died in our study. Reduced membrane permeability, increased efflux and Class B and D carbapenemase production are concurrent resistance mechanisms in *A. baumannii*. Consequently, CFD was the unique option among the novel antibiotics for CRAB infections (Delgado-Valverde et al., 2020; Falcone et al., 2022). Once again, heteroresistance to CFD has been described in *A. baumannii*, however its link with increased mortality in CARB infections treated with this antibiotic remains questionable (Bassetti et al., 2020; Karakonstantis et al., 2020).

Finally, regardless of the gram-negative bacteria targeted, clinicians in charge of patients requiring CFD should be aware that some concerns have been raised in CFD Antimicrobial Susceptibility Testing. In any case, testing of isolates prior to CFD use is mandatory and result interpretation of these tests requires expertise in the field (Simner and Patel, 2020).

We reported non-severe adverse events after 5 antibiotic courses (17%). According to previous published study, CFD, IMI-REL and CAZ-AVI-ATM have good tolerance profile, better than ancient combination therapies based on colistin, tigecycline and fosfomycin (Bassetti et al., 2020; Brown et al., 2020; Falcone et al., 2021; Falcone et al., 2022). Thanks to good tolerance, large therapeutic margin, and sufficient stability after reconstitution, PK/PD optimization could allow to increase success rate of last resort antibiotics (Loeuille et al., 2022).

We reported the feasibility of CFD administration by continuous infusion in two patients. Regarding CFD concerns about heteroresistance and treatment failure, continuous infusion appears to be a promising alternative to improve resistance suppression and treatment success rates (Karakonstantis et al., 2022). We reported therapeutic success with continuous infusion of CFD for the treatment of a meningitis (Meschiari et al., 2021; Luque-Paz et al., 2022), but also in bone and joint infections (Siméon et al., 2020; Mabayoje et al., 2021; Simner et al., 2022).

In the same line, it has been estimated using a hollow-fiber infection model that CAZ-AVI-ATM as continuous infusions resulted in maximal bacterial killing and resistance suppression over 7 days (Lodise et al., 2020). However, clinical data are limited to a case report (Cowart and Ferguson, 2021). We reported herein a novel case of CAZ-AVI-ATM administration by continuous infusion associated with a therapeutic success in a patient with a nosocomial pneumonia due to NDM-producing *K. pneumoniae* (Loeuille et al., 2022).

We must acknowledge some limitations to our study. Our conclusions are limited by the relatively small size of the cohort and by the retrospective and single center design of the study, which could induce bias in data collection and results interpretation. Particularly, our estimation of therapeutic success rates may have been flawed due to the small number of included patients, particularly for IMI-REL and CAZ-AVI-ATM. However, case series of patients treated with last resort antibiotics are rare and rates reported herein are within the same range of those previously reported (Falcone et al., 2021; Meschiari et al., 2021; Timsit et al., 2022a). Lastly, despite wgMLST highlighted the large diversity of isolates with DTR in the present study, our results could not be generalized to all countries/hospital due to the variability in geographical distribution of bacterial infections and AMR.

To conclude, last resort beta-lactam antimicrobials use in real-life settings was associated with relatively low rates of microbiological and clinical failure, recurrence of infection and death, at 33%, 30%, 20% and 23%, respectively. Thus, the probability of infection-free survival was 48.4% CI95% [33.2-70.5] 90-day after antibiotic initiation. Moreover, the rate of adverse events was under 20%. Taking into account the severity of the diseases and patients’ conditions, last resort beta-lactam antimicrobials were safe and efficient therapeutic options for treatment of severe infections related to DTR-*P. aeruginosa* (CFD and IMI-REL), DTR-*S. maltophilia* (CFD and CAZ-AVI-ATM), CRAB (CFD), and NDM-producing-*K. pneumoniae* (CAZ-AVI-ATM and CFD). Our results underlined the difficulties encountered in the management of bacteria with DTR infections. Nevertheless, they also highlighted care opportunities offered by new antibiotics for patients no further therapeutic option was available until recently.

## Data availability statement

The raw data supporting the conclusions of this article will be made available by the authors, without undue reservation.

## Ethics statement

The studies involving human participants were reviewed and approved by Institutional Review Board of Nimes University Hospital. Written informed consent for participation was not required for this study in accordance with the national legislation and the institutional requirements.

## Author contributions

RL and AS contributed to the study conception and design. Material preparation, data collection and analysis were performed by RL, PL-L, RD, CG-V and AP. The first draft of the manuscript was written by RL and PL-L, then AP and AS commented on previous versions of the manuscript. All authors contributed to the article and approved the submitted version.

## Conflict of interest

The authors declare that the research was conducted in the absence of any commercial or financial relationships that could be construed as a potential conflict of interest.

## Publisher’s note

All claims expressed in this article are solely those of the authors and do not necessarily represent those of their affiliated organizations, or those of the publisher, the editors and the reviewers. Any product that may be evaluated in this article, or claim that may be made by its manufacturer, is not guaranteed or endorsed by the publisher.
